# mSphere of Influence: How a gut microbiota study reshaped my thinking on tick-borne pathogens

**DOI:** 10.1128/msphere.00213-26

**Published:** 2026-05-20

**Authors:** Xin-Ru Wang

**Affiliations:** 1 Department of Microbiology and Immunology, Upstate Medical University 12302, Syracuse, New York, USA; 2 SUNY Center for Vector-Borne Diseases, Upstate Medical University 12302, Syracuse, New York, USA; 3 Institute for Global Health and Translational Sciences, Upstate Medical University 12302, Syracuse, New York, USA; Virginia-Maryland College of Veterinary Medicine, Blacksburg, Virginia, USA

**Keywords:** gut microbiome, tick-borne pathogens, host-pathogen interactions, innate immunity

## Abstract

Xin-Ru Wang works in tick biology and intracellular bacterial pathogenesis. In this mSphere of Influence article, she reflects on how “Gut microbiota of the tick vector *Ixodes scapularis* modulate colonization of the Lyme disease spirochete” by Narasimhan et al. (2014) reshaped her understanding of vector competence by placing pathogen colonization within the ecological context of the tick microbiome. Her laboratory studies *Rickettsia*-tick cell interactions, including autophagy, apoptosis, and innate immune signaling. Here, she examines how this ecological framework extends to the intracellular level, where pathogenic *Rickettsia* may encounter cellular environments already shaped by resident endosymbionts.

## COMMENTARY

When I entered the tick biology field in 2018, Narasimhan et al. (1) was already on my reading list. I read it, took notes, and moved on, as new researchers do when they are trying to rapidly build foundational knowledge. I understood the main findings. I filed the paper away as “important” and moved on, continuing to build the framework I thought I needed: one pathogen, one host, and one relationship at a time. My laboratory’s focus was narrow by design. We worked on *Rickettsia*: how it invades tick cells, how it subverts autophagy, whether it manipulates apoptosis to extend its intracellular residence, and how tick innate immune signaling shapes infection. These are bilateral questions—clean and tractable. They suited a young laboratory trying to establish itself in a well-defined system. It was only years later, when I began to place *Rickettsia* within the broader ecology of the tick—to ask not just how the bacterium behaves within a cell but also what kind of environment that cell inhabits—that I returned to the 2014 study. Reading it again, I realized something I had missed the first time: I had absorbed the conclusions without fully understanding how the paper had reshaped the question itself. Narasimhan et al. had not just reported a finding about *Borrelia* and tick microbiota. They had redrawn the map, expanding the system from a bilateral interaction to an ecological community. The paper did not change a specific experiment: it changed the way I was asking questions. Before, I had viewed the tick as a controlled system, one in which a pathogen and its arthropod host negotiated their interaction. Reading it again, I understood that this system was already inhabited. The pathogen was not entering an empty room. It was entering a community, with its own residents, rules, and ecology. That realization has since become foundational to how I think about tick-borne disease—and about *Rickettsia* in particular.

The experimental logic of the paper was elegantly simple. Narasimhan and colleagues treated larval *Ixodes scapularis* ticks with gentamicin to perturb their gut microbiota and then assessed the ticks’ ability to acquire *Borrelia burgdorferi* during a blood meal. Microbiota disruption significantly impaired spirochete colonization. The mechanism ran through JAK-STAT signaling: disruption of the microbial community altered the expression of STAT-regulated genes, including peritrophin-1, a structural component of the peritrophic matrix lining the tick midgut. When that barrier was compromised, *Borrelia* colonization suffered. The microbiota was not a bystander. It was actively maintaining the conditions that governed whether a pathogen could establish itself at all. The conceptual significance of this finding, however, needs careful framing. In mammalian gut biology, the resident microbiota typically acts as a barrier against pathogen invasion, a phenomenon termed colonization resistance (2). However, Narasimhan et al. found the opposite relationship: in *Ixodes*, the indigenous microbiota promoted *Borrelia* acquisition. Disrupting it did not facilitate infection, as a colonization resistance model would predict, but blocked it. It demonstrates that the microbiome’s impact on vector competence is not simply a transposition of mammalian principles into an arthropod—the outcomes can be fundamentally different, and the directionality must be established empirically for each system. The shared principle, however, is that the resident microbial community determines whether a pathogen can establish itself: not a passive backdrop but an active gatekeeper.

The research followed. Abraham et al. (3) introduced a key shift: rather than asking how the microbiome shapes pathogen colonization, they showed that *Anaplasma phagocytophilum* actively manipulates the tick microbiome to its own advantage, inducing tick expression of an antifreeze glycoprotein (IAFGP) that selectively disrupts gram-positive bacterial biofilms and compromises the peritrophic matrix barrier. The direction of influence was no longer one-way—the pathogen can also reshape the microbiome. Later, Duron et al. (4) showed that ticks depend on bacterial endosymbionts as obligate providers of B vitamins, making the resident microbial community fundamental to tick fitness itself. Together, these studies establish that tick immune physiology, resident microbial community, and invading pathogens form a three-way interaction—each shaping the others. None of these can be fully understood in isolation.

This model is directly relevant to my own work, but it cannot be applied without modification. Unlike *Borrelia*, which colonizes the tick midgut lumen before disseminating, *Rickettsia* invades midgut epithelial cells upon acquisition and subsequently disseminates via the hemolymph to salivary glands and ovaries, maintaining a predominantly intracellular lifestyle. The gut lumen dynamics central to the study by Narasimhan et al. are therefore unlikely to represent the primary interface for *Rickettsia*-microbiome interactions. *Rickettsia* offers a unique opportunity to test this adapted model. The genus spans a remarkable biological range: from ancient endosymbionts, vertically transmitted and tightly integrated into tick physiology, to highly pathogenic species responsible for Rocky Mountain spotted fever. In some tick populations, endosymbiotic and pathogenic *Rickettsia* coexist, potentially within the same cell. What happens when they coexist within the same cellular environment remains largely uncharacterized. My laboratory studies how tick cells respond to infection, through autophagy, apoptosis, and innate immune signaling. These are the same processes that a pathogenic *Rickettsia* must navigate to establish itself intracellularly. If an endosymbiont is already present, however, the cellular environment may be altered before the pathogen arrives. We have begun to test this directly; detailed findings will be reported elsewhere.

Key questions remain. Mechanistic studies of microbiome-pathogen interactions in ticks have focused largely on the midgut; work on salivary glands and ovaries remains limited. For pathogens such as *Rickettsia* that disseminate systemically, critical interactions may occur in tissues whose microbial communities are still poorly defined. Within the genus *Rickettsia*: do endosymbionts and pathogens compete for intracellular resources? Does a resident endosymbiont alter the intracellular immune environment that a pathogenic *Rickettsia* must navigate? These questions are central to understanding rickettsial disease emergence, yet they have not been systematically addressed. At the translational level, Utarini et al. (5) demonstrated that *Wolbachia*-based microbiome manipulation can be an effective vector control strategy. Whether analogous approaches can be extended to ticks, with their longer life cycles, distinct reproductive biology, and broader pathogen diversity, remains an open question.

I began research in this field asking how a single bacterium subverts a single cell. I have not abandoned that question, but I now approach it differently. A pathogen that enters a tick does not encounter an empty system; it enters an established community shaped by resident microbes. Understanding that community is not a distraction from the biology of infection. It is the biology of infection, viewed at the appropriate scale—one that requires integrating microbial ecology, vector immunology, cell biology, and evolutionary biology. I am grateful to Narasimhan et al. (1) for what is, on its surface, a study of *Borrelia* in larval *Ixodes* ticks. It has had a disproportionate influence on a laboratory that works on neither organism. That is the hallmark of an important paper: it does not merely answer a question; it teaches you to ask better ones.
